# Anti-Psoriatic Activity of Black, Green and White Tea Extracts from Southeastern China

**DOI:** 10.3390/molecules29061279

**Published:** 2024-03-13

**Authors:** Lanyue Zhang, Zeting Huang, Jie Xuan, Lu Yang, Tiantian Zhao, Weihua Peng

**Affiliations:** 1 Guangzhou Zhongzhuang Meiye Cosmetics Co., Ltd., Guangzhou 510006, China; huangzeting@hanhoo.com (Z.H.); xuanjie@hanhoo.com (J.X.); yangpeishan23@hotmail.com (L.Y.); 2 Guangdong Provincial Key Laboratory of Plant Resources Biorefinery, School of Biomedical and Pharmaceutical Sciences, Guangdong University of Technology, Guangzhou 510006, China; 3 Sericulture and Agri-Food Research Institute, Guangdong Academy of Agricultural Sciences, Key Laboratory of Functional Foods, Ministry of Agriculture and Rural Affairs, Guangdong Key Laboratory of Agricultural Products Processing, Guangzhou 510610, China; fettzhao1989@163.com; 4 Department of Food Science, Rutgers University, New Brunswick, NJ 08901, USA

**Keywords:** psoriasis, tea, inflammation, ethanol extract, component analysis

## Abstract

Psoriasis is a common chronic inflammatory disease, but most of its current treatments come with a high risk of side effects. As one of the world’s top three beverages, tea has a traditional history of being used as a treatment for skin conditions due to its high safety profile, anti-inflammatory and other properties. In this study, we investigated the anti-psoriasis effects of ethanol extracts of black tea, green tea and white tea from southeastern China. The compositions of the tea extracts (TEs) were first determined by UPLC-Q-Exactive-Orbitrap MS and then genetic analysis, antibacterial, anti-inflammatory, and immunocompetence assays were performed. Imiquimod was used to establish a mouse model of psoriasis-like dermatitis and treating with the extracts to examine their efficacy. A total of 88 chemical components, mainly phenols and organic acids, were identified from the TEs. These TEs ameliorated skin damage and they all reduced the expression of cytokines IL-17 and TNF-α. By analyzing the genes, TEs may affect the inflammatory signaling pathway by regulating the metabolic changes. In addition, TEs can significantly scavenge ROS, NO, and inhibit cellular inflammation. In conclusion, this study examined the inhibitory effects of three TEs on psoriasis and their potential as nutritional supplements for the treatment of skin inflammation.

## 1. Introduction

Psoriasis is a common chronic immune-mediated inflammatory skin disease that affects 2% to 4% of the population, an estimated 12.51 billion people worldwide [1,2]. It seriously affects physical health and quality of life [3]. Approximately 80–90% of clinical psoriasis cases are psoriasis vulgaris (PsV). PsV is characterized by erythematous, well-defined, pruritic plaques covered by silvery scales. It affects the scalp, trunk and extensor surfaces of the extremities [4]. Psoriasis is an IL-17A and IL-23 cytokine-driven autoimmune skin disease mediated by the adaptive and innate immune systems [5]. All current treatments for psoriasis revolve around the use of synthetic drugs, which can lead to serious adverse side effects [6]. In contrast, natural products are becoming widely used in psoriasis treatment because of their limited side effects [7,8,9].

As a member of the natural products, tea has an attractive aroma, good taste and health-promoting effects, which makes it as one of the top three beverages in the world. Tea is an infusion of the leaves of *Camellia sinensis* (L.) O. Ktze. and contains many bioactive components [10,11]. Tea has a variety of pharmacological functions such as anti-allergic, anti-depressant, antioxidant and other abilities [11,12,13]. The tea tree originated in China and is now widely grown in the Southeast Asian region and in Central African countries, with slight variations in the tea produced in different places [14].

The six main types of tea in China include green tea (GT), black tea (BT) and white tea (WT). GT is a partially fermented tea beverage that is prepared when the fresh leaves are processed quickly to prevent “fermentation”. GT inhibits angiogenesis and possesses antibacterial potential [15,16]. Black tea is made by “fermenting” the fresh tea leaves by crushing and drying them before final processing. During the “fermentation” process, some catechins combine to form complex theaflavins and other undefined flavonoids that provide black tea beverages with their unique flavor and color [10]. Polyphenols such as theaflavins and theobromine, as well as catechins, are mainly responsible for the antioxidant effects of black tea as the main components of black tea [17]. White tea has no fermentation stage and the basic process is withering, sun-drying or drying, making it a simple process. It has been shown to have antioxidant and preventive effects against chronic diseases [18,19].

Herein, the ethanol extracts of three teas (green tea, black tea, and white tea) harvested from China were studied. Their main components were determined by the UPLC-Q-Exactive-Orbitrap MS method, and their antibacterial effects, and effects on imiquimod (IMQ)-induced psoriasis mouse model and cells were investigated to evaluate their mechanism and effects in the treatment of psoriasis and to determine their medicinal potential for inflammatory diseases.

## 2. Results

### 2.1. Experimental Result

#### 2.1.1. Chemical Composition of Ethanol Extract of Three Kinds of Tea

The components of the three kinds of tea were roughly the same, but black tea had the biggest difference in composition. Table 1 shows the specific ingredients of the three teas. Ethanol extract of *C. sinensis* was obtained by final analysis. Among them, the main components of green tea were 1-stearyl glycerol (24.48%), 2,2′-methylene double (4-methyl-6-tert-butyl phenol) (6.88%), stearamide (5.26%), trehalose (5.25%), and erucamide (4.20%). The main components of black tea are caffeine (34.37%), 1-stearyl glycerol (30.98%), 2,2′-methylene double (4-methyl-6-tert-butylphenol) (6.15%), sineramide (4.63%), and d-(−)-quinic acid (3.48%). The main components of white tea were 1-stearyl glycerol (40.15%), caffeine (17.96%), 2,2′-methylene double (4-methyl-6-tert-butylphenol) (6.15%), erucamide (5.89%), and bis-(4-ethylbenzylidene) sorbitol (4.58%). 

#### 2.1.2. Antibacterial Ability

**Figure 1:**
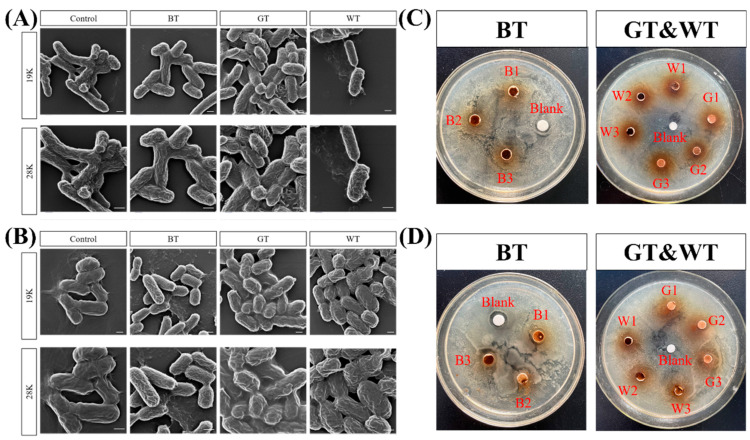
Effects of three alcoholic extracts of tea on surface morphological changes of bacteria. (**A**) Morphological effects of three tea extracts on *E. coli*. (**B**) Morphological effects of three tea extracts on *S. aureus*. (**C**) Antibacterial effect of three TEs on *E. coli*. In GT&WT, the upper left is WT, and the lower right is GT. (**D**) Antibacterial effect of three tea extracts on *Staphylococcus aureus*. In GT&WT, WT is at the lower left and GT is at the upper right. Scale bar: 500 nm.

As can be seen from Figure 1A, *Escherichia coli* in normal growth state was oval and rod-shaped with full surfaces. The three TEs groups all affected the morphology of *Escherichia coli* to some extent. GT extract had the greatest impact on the morphology of *Escherichia coli*, forming a short rod-shape, indicating that the three TEs could affect cell division by affecting the morphology of *Escherichia coli*, thereby achieving antibacterial effects. As shown in Figure 1B, *Staphylococcus aureus* in normal growth state is a round cake with smooth and full surface. The three TEs groups all affected the morphology of *S. aureus* to varying degrees, among which GT extract had the greatest effect on the morphology of *S. aureus*, indicating that the three TEs could affect the cell division of *S. aureus* by affecting the morphology of *S. aureus*. According to the experimental results, GT has the best antibacterial effect. Figure 1C,D show the results of bacteriostatic zone. Table 2 shows that for *E. coli*, the bacteriostatic effect of three kinds of tea is similar, BT has the best effect, and for *S. aureus*, BT has the best bacteriostatic effect.

#### 2.1.3. Animal Experiments

##### Apparent Skin Evaluation and HE Staining

**Figure 2:**
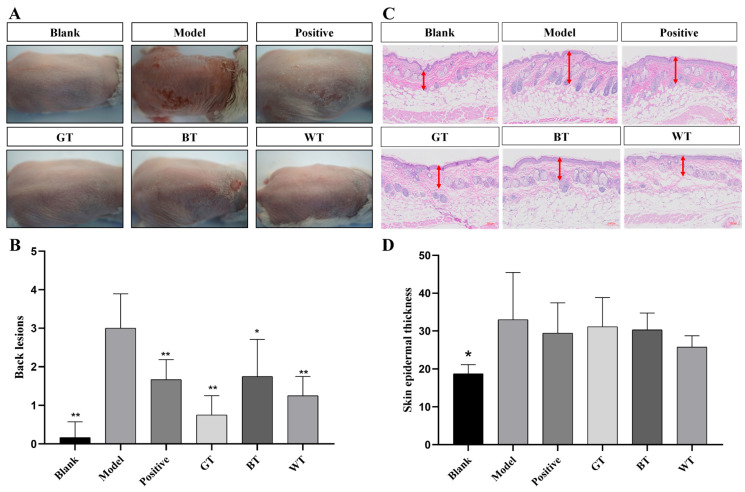
Apparent skin evaluation and testing of skin HE staining results. (**A**) Skin repair in modeling mice. (**B**) Significance analysis of apparent skin assessment. (**C**) Test skin HE staining results. (**D**) Significance analysis of test skin HE staining results. Data plotted as mean ± standard deviation (n = 3 animals/group). The length of the arrow indicates the skin epidermal thickness. Compared with model group, * *p* < 0.05, ** *p* < 0.01. Scale bar: 100 μm.

The PASI score is often used to assess the severity of psoriasis. As shown in Figure 2A,B, for the therapeutic effect of modeling mice, WT, GT and Positive groups showed the best significant effect (*p* < 0.01), while black tea group had good effect (*p* < 0.05). The histological analysis of the tested skin was shown in Figure 2C,D. The epidermal hyperplasia of the positive group, all tea extract groups and blank group was lower than that of the model group, indicating that the local application of the three TEs could effectively inhibit the epidermal hyperplasia caused by allergic dermatitis. The WT extract group showed better inhibitory effect.

##### Skin Toluidine Blue Staining

**Figure 3:**
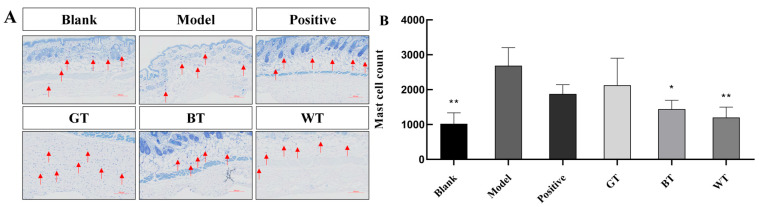
The results of skin toluidine blue staining were tested. (**A**) Staining results. (**B**) Significance analysis of toluidine blue staining results. Data plotted as mean ± standard deviation (n = 3 animals/group). Arrows indicate the mast cells observed. Compared with model group, * *p* < 0.05, ** *p* < 0.01. Scale bar: 100 μm.

As shown in Figure 3, the number of mast cells in the dermis in the blank group was significantly reduced compared to the model group. In the drug group, the degree of mast cell infiltration in mice treated with extracts of WT and BT was significantly lower than in the model group, and the degree of mast cell infiltration was lowest in the skin tissues of mice treated with WT extract. This suggests that WT extract inhibits psoriasis-induced skin inflammation.

##### Immunohistochemistry

**Figure 4:**
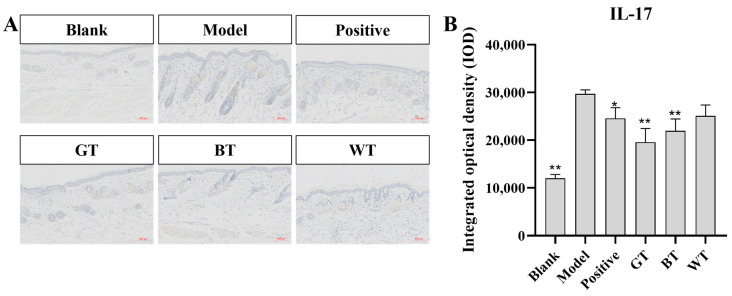
Immunohistochemical results. (**A**) Immunohistochemical results of IL-17. (**B**) Significance analysis of immunohistochemical results of IL-17. Data plotted as mean ± standard deviation (n = 3 animals/group). Compared with model group, * *p* < 0.05, ** *p* < 0.01. Scale bar: 100 μm.

As shown in Figure 4, compared with model group, the expression of pro-inflammatory cytokine IL-17 in blank group was significantly increased (*p* < 0.01), and the expression of IL-17 in BT extract group and GT extract group was significantly decreased compared with model group (*p* < 0.01). The positive group could inhibit the expression of IL-17 (*p* < 0.05).

**Figure 5:**
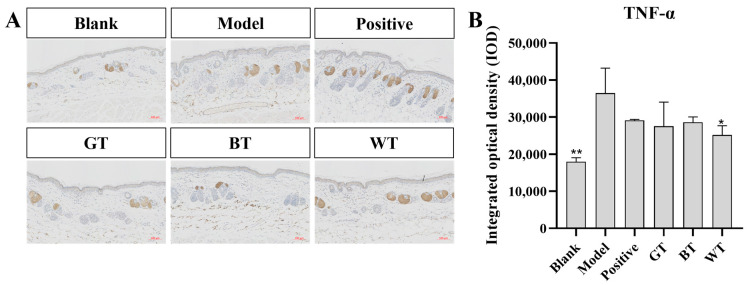
Immunohistochemical results. (**A**) Immunohistochemical results of TNF-α. (**B**) Significance analysis of TNF-α immunohistochemical results. Data plotted as mean ± standard deviation (n = 3 animals/group). Compared with model group, * *p* < 0.05, ** *p* < 0.01. Scale bar: 100 μm.

As shown in Figure 5, compared with blank group, the expression of pro-inflammatory cytokine TNF-α in model group was significantly increased (*p* < 0.01), while that in WT extract group was significantly decreased (*p* < 0.05).

##### Analysis of Differentially Expressed Genes and Their Expression Levels

**Figure 6:**
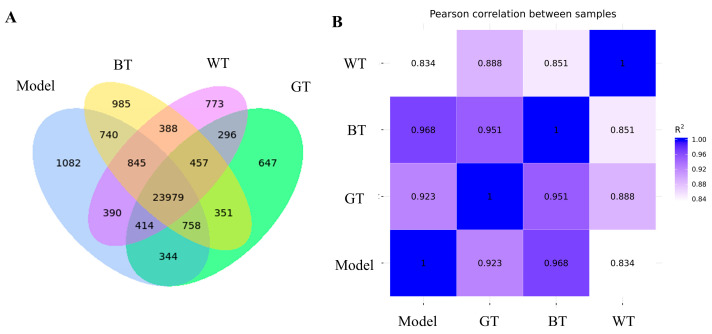
(**A**) Venn diagram of co-expressed genes; (**B**) sample correlation.

Pearson correlation coefficient (R) was selected as the evaluation index of biological repeat correlation in this paper. The closer the absolute value of R is to 1, the stronger the correlation between the two duplicate samples, and this is the key criterion. In Figure 6A, a total of 23,979 co-expressed genes were analyzed, and the most co-expressed genes were found in the BT group and the model group among the treatment groups. This was also demonstrated in Figure 6B, with the highest R2 value of 0.968 for the BT group versus the model group.

Genetic screening standard default differences *p* < 0.05, | | log 2 FC > 1, the differences between the treatment group and model group expressed genes (DEGs) were compared; the Venn diagram shows each group and the only DGEs. Overall, 2424 differentially expressed DEGs were identified, a total of 28 DEGs. The number of differentially expressed genes in different treatment groups and model groups was statistically significant.

**Figure 7:**
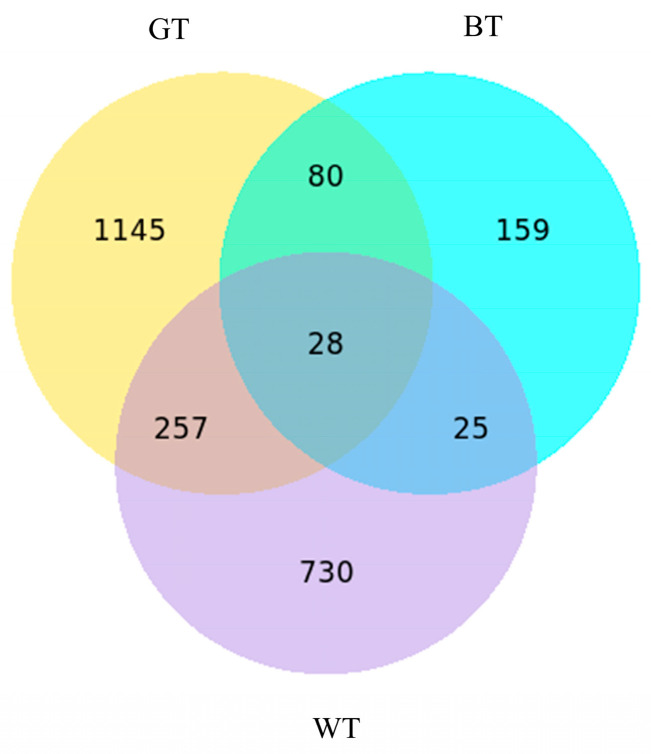
A ternary Venn diagram of three differential groups. All groups are compared with the model group.

The change in gene expression ratio was shown as the horizontal axis of the volcano map, and the level of gene significance was shown as the vertical axis. The up-regulated differential genes are represented by red dots, the down-regulated differential genes are represented by green dots, and the non-differentially expressed genes are represented by blue dots. As can be seen from Figure 7, the GT group had the most up-regulated and down-regulated genes, and the WT group also had significant down-regulated genes.

**Figure 8:**
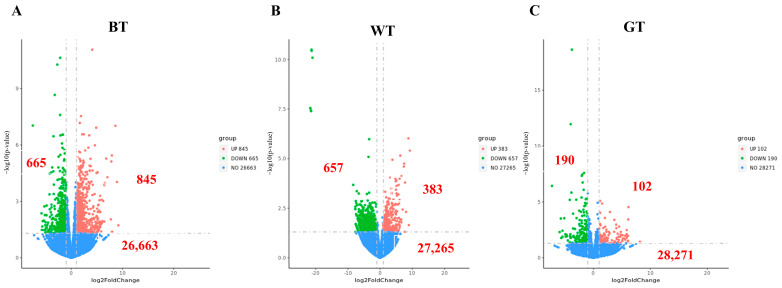
Volcanic maps of (**A**) model vs. GT, (**B**) model vs. WT, (**C**) model vs. BT.

The total number of genes detected in differential groupings and the number of significantly up- and down-regulated differential genes are shown by volcano plots. Fold change in gene expression is represented as the volcano plot horizontal axis, and gene significance levels are represented as the vertical axis. Up-regulated differentially expressed genes are represented by red dots, down-regulated differentially expressed genes are represented by green dots, and non-differentially expressed genes are represented by blue dots. As can be seen from Figure 8, the GT group had the most up- and down-regulated genes compared to the model group, and the WT group also had significant down-regulated genes.

**Figure 9:**
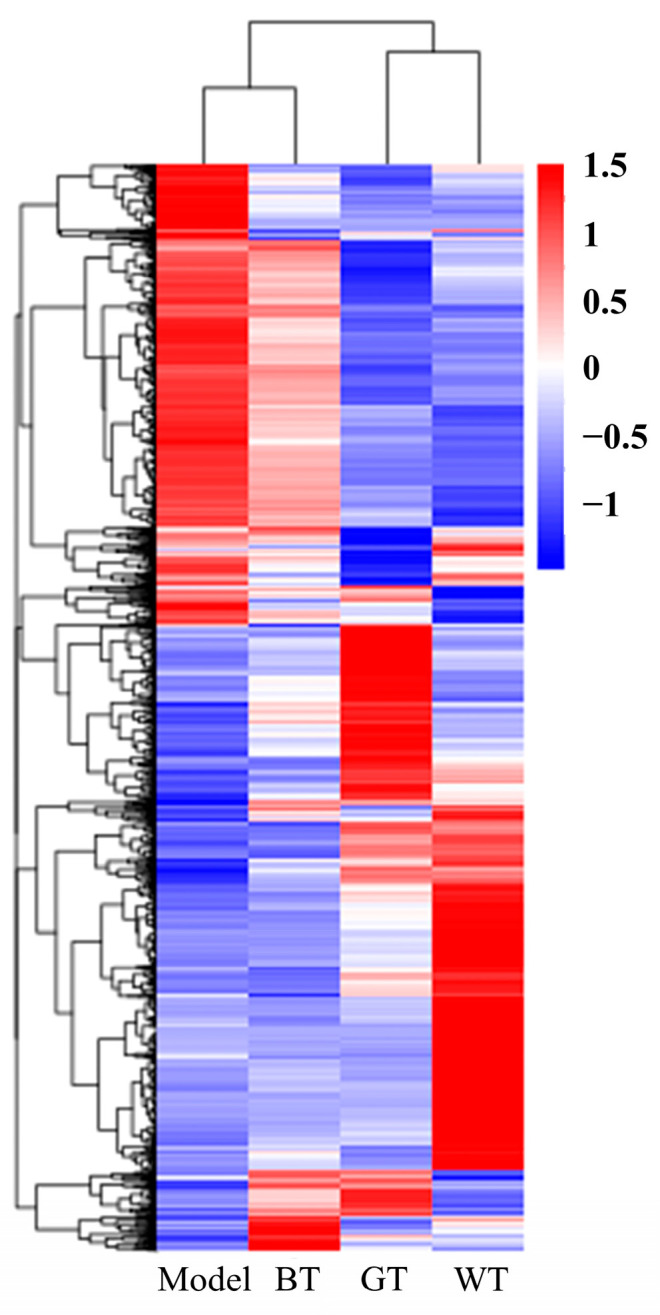
Cluster heat map.

As can be seen from Figure 9, the genes significantly expressed in the model group were not significantly expressed in the treatment group, especially in the WT treatment group.

##### Gene Function Analysis

**Figure 10:**
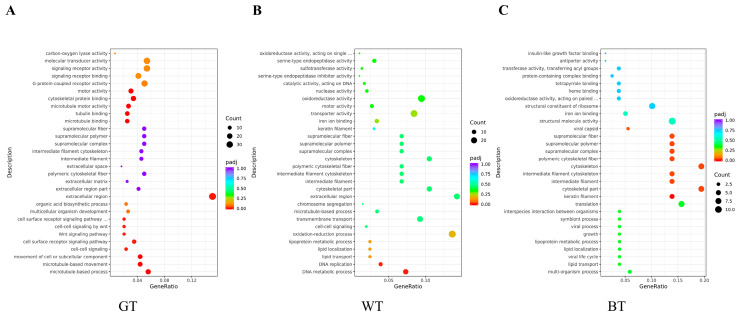
Genetic ontological functions of (**A**) model vs. GT group, (**B**) model vs. WT group, and (**C**) model vs. BT group.

One of the international standard gene function classification systems is Gene Ontology (GO). GO consists of three components, biological processes, molecular functions and cellular components. As can be seen from Figure 10, GO gene enrichment is mainly manifested in signaling receptor activity (GT group), REDOX process (WT group) and cytoskeleton (BT group). These gene enrichment pathways were abundant and significantly enriched.

**Figure 11:**
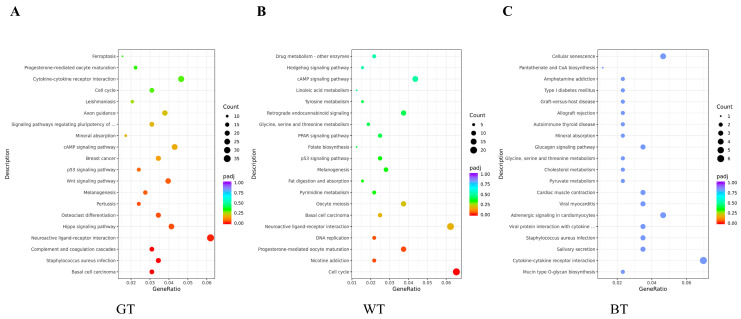
KEGG enrichment in (**A**) model vs. GT group, (**B**) model vs. WT group, and (**C**) model vs. BT group.

In Figure 11 KEGG was seen to be enriched in 20 signaling pathways in each control group, which were mainly related to signaling pathways such as inflammatory response, immune regulation, hormone synthesis and metabolism, and vitamin regulation. The active ingredients of the three tea alcoholic extracts could modulate multiple signaling pathways through a variety of target proteins such as TNF-α, IL-17, FLG, Cytokeratin 17, IL-23, STAT3, FLG, IFN-γ, IL-22, etc., and thus attenuate or inhibit psoriasis. Specifically, compared with the model group the GT and WT groups were enriched in common neuroactive ligand-receptor interaction signaling pathways, while the BT group was closely associated with signaling pathways such as cytokine-cytokine receptor interaction. The present results will provide a theoretical basis and information foundation for further investigation of the potential inhibitory effects of the three groups of TEs on psoriasis.

#### 2.1.4. Cell Experiment

##### MTT Experiment Results

**Figure 12:**
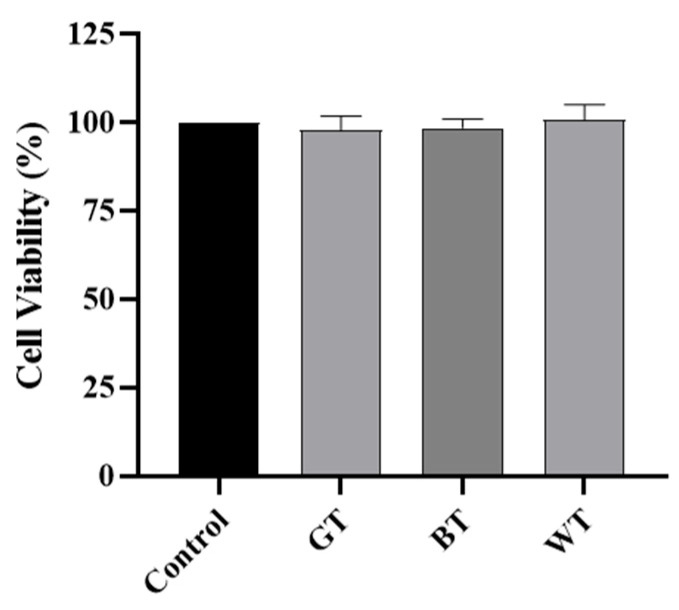
Cell viability test of three tea extracts. Data plotted as mean ± standard deviation.

As shown in Figure 12, compared with the control group, the cell vitality of the three tea extracts after treatment was almost at the same level, indicating that the three tea extracts were not cytotoxic and could be used for treatment.

##### ROS Release

**Figure 13:**
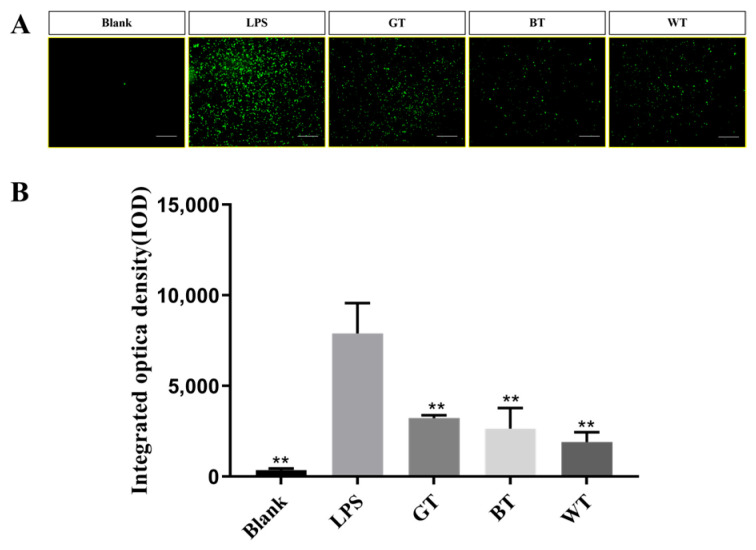
Effects of extracts on the release of RAW264.7 ROS in macrophages. Data plotted as mean ± standard deviation. (**A**) Photographed cell fluorescence images. (**B**) Results of optical density measurements. Compared with the LPS group, ** *p* < 0.01. Scale bar: 200 nm.

As shown in Figure 13, under the influence of lipopolysaccharide (LPS), reactive oxygen (ROS) release in macrophages was significantly increased compared with the blank group (*p* < 0.01), indicating that ROS release in macrophages was significantly increased after LPS induced cell inflammation model. After different extracts were given to co-culture cells, the ROS release of macrophages in all groups was significantly decreased compared with LPS (model group) (*p* < 0.01), indicating that the extract group in this study can significantly reduce the inflammatory response and intracellular ROS release caused by LPS, and can better resist the inflammatory stimulus caused by LPS.

##### Determination of NO Release

**Figure 14:**
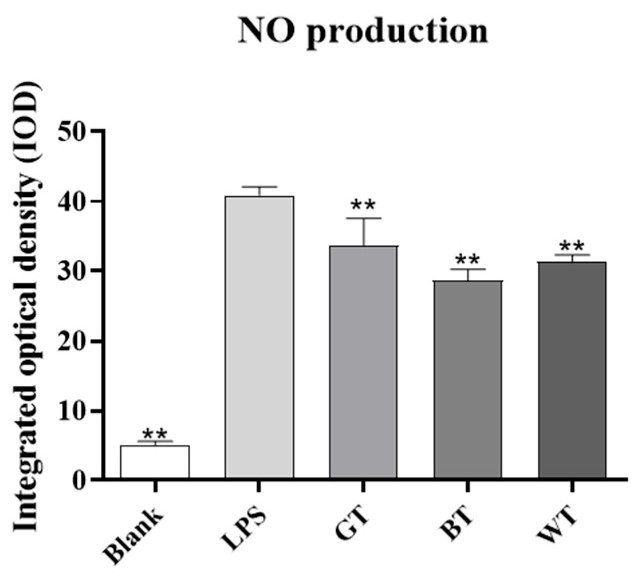
Effects of extracts on the release of RAW264.7 NO in macrophages. Data plotted as mean ± standard deviation. Compared with the LPS group, ** *p* < 0.01.

As shown in Figure 14, after LPS stimulation, the amount of nitric oxide (NO) released by macrophages increased significantly (*p* < 0.01). The release of NO from macrophages in other groups decreased compared with LPS model group.

##### Migration Scratch

**Figure 15:**
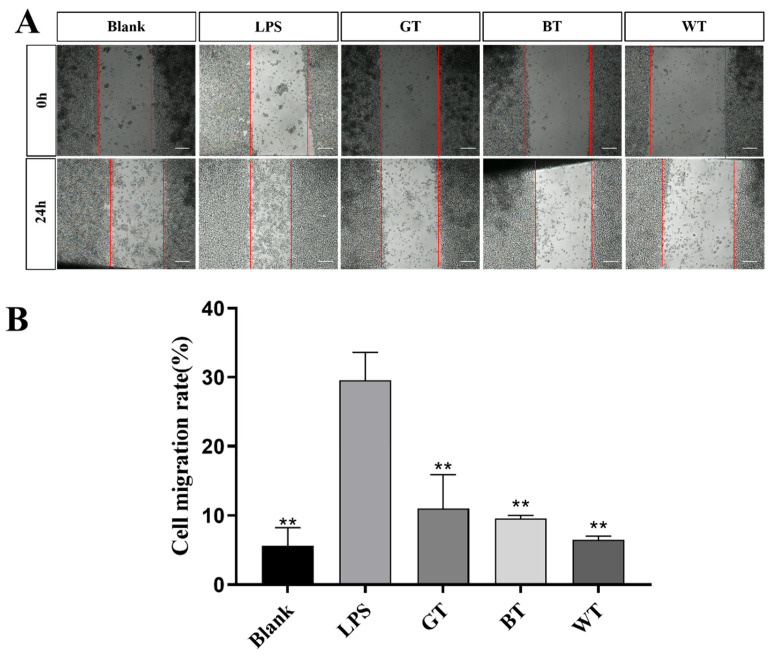
(**A**) Cell migration and (**B**) cell mobility of three tea extracts. Data plotted as mean ± standard deviation. The red line shows the outline of the scratch. Compared with the LPS group, ** *p* < 0.01. Scale bar: 200 μm.

As shown in Figure 15, compared with the blank group, cell mobility in the model group was significantly increased, indicating that LPS in the model group caused cellular inflammation. Compared with the model group, cell mobility of the three tea extracts was significantly reduced.

##### Phagocytic Neutral Red Experiment

**Figure 16:**
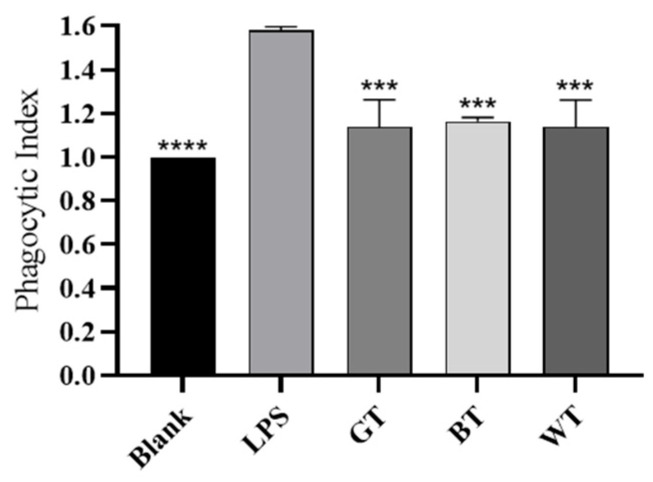
Phagocytosis index of three tea extracts. Data plotted as mean ± standard deviation. Compared with the LPS group, *** *p* < 0.001, **** *p* < 0.0001.

As shown in Figure 16, when LPS alone stimulated macrophages of mice for 24 h, the phagocytic index of the LPS group was significantly higher than that of the blank group, indicating that LPS stimulation can significantly activate macrophages and improve their phagocytic ability. The phagocytic ability of phagocytic cells was significantly down-regulated in all experimental groups after 24 h treatment with ethanol extracts from other kinds of tea leaves.

##### ELISA Experiment Results

**Figure 17:**
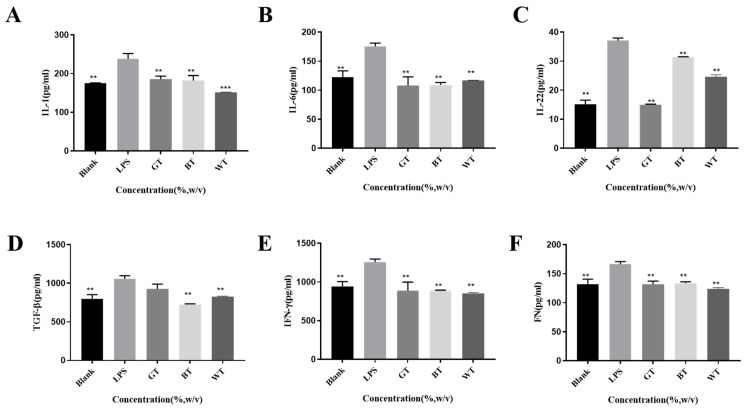
Detection of the associated protein factors IL-1 (**A**), IL-6 (**B**), IL-22 (**C**), TGF-β (**D**), IFN-γ (**E**), and FN (**F**). Data plotted as mean ± standard deviation. Compared with the LPS group, ** *p* < 0.01, *** *p* < 0.001.

Analyzing the therapeutic effects of psoriasis at the molecular level is also an important strategy [20]. The effect of these three tea extracts on the expression of six cytokines (IL-1, IL-6, IL-22, TGF-β, IFN-γ and FN) associated with this disease was investigated (Figure 17). The alcoholic extracts of *Camellia sinensis* processed by three different processes can inhibit and reduce the cytokines causing inflammation, and have anti-inflammatory effects. Among them, the alcohol extract of white tea has down-regulation effect on the above six factors, and the inhibition effect is more balanced. Green tea extract showed the worst inhibitory effect on TGF-β and the best inhibitory effect on IL-22 compared with the other two tea extracts. The effect of black tea extract on IL-22 inhibition was worse than that of the other two kinds of tea, and it could play a good inhibitory effect in other aspects.

## 3. Discussion

By the method of UPLC-Q-Exactive-Orbitrap MS, it was found that the components of white tea and green tea were similar, while the components of black tea were quite different. It could be concluded that the differences were caused by the processing technology [21,22,23]. The chemical composition of the extracts often has a great impact on the biological activity of the extracts. As a natural sugar, trehalose is found in high levels in GT and WT extracts (5.25% and 2.74%, respectively) and can inhibit photodamage induced by UVB rays [24]. Caffeine is abundant in the alcoholic extracts of BT and WT (34.37% and 17.96%, respectively) and possesses excellent antioxidant and anti-inflammatory properties, which may be beneficial in the treatment of inflammatory diseases [25].

There are many people affected by psoriasis globally and natural product teas have been little used and studied in this area [9], especially these three tea extracts (GT, BT and WT) are also proposed for the first time to synthesize and compare the therapeutic effects on psoriasis from multiple perspectives. 

Progress in the treatment of psoriasis can be understood by examining the interactions between the immune system and the skin microbiome [26]. Chang et al. demonstrated that *Staphylococcus* spp. are in high abundance in psoriatic plaques and that *S. aureus* is a potential contributor to the pathogenesis of psoriasis [27]. Our study found that GT had the best inhibitory effect on *S. aureus*, with an inhibitory circle diameter of only 1.2 ± 0.69 mm (in Table 2), and disruption of bacterial morphology. Many studies have also demonstrated the link between psoriasis and intestinal flora dysbiosis, which is associated with metabolic, immune and protective functions of the body [28,29]. One study showed significant enrichment of *E. coli* in psoriasis patients compared to healthy individuals [30]. All three TEs were found to cause changes in bacterial surface morphology, with GT causing the most pronounced growth inhibition of *E. coli*. Taken together, the results of the bacterial experiments suggest that GT can best regulate the bacterial flora to have therapeutic potential for psoriasis.

The PASI score is often used to reflect the degree of change in the appearance of a skin lesion, with the score positively correlating with the extent of the lesion. It can be visualized that IMQ induced skin indicates visible and obvious scaling. The apparent smoothness and integrity of the skin in the treated group compared to the model group can be clearly seen in Figure 2A. The skin was also significantly smoother and more intact in the model group than in the treated group. In addition, a reduction in the thickness of the skin in the treatment group was seen as measured by HE staining. Mast cells are very important regulators of the development of psoriasis and an increase in infiltrating mast cells exacerbates the development of inflammation [31,32]. We analyzed the effect of TEs on this by toluidine blue staining and found a significant reduction in mast cells in the BT and WT groups compared to the model group. 

The IL-17 pathway-mediated immune cascade response plays a key role in the pathogenesis of psoriasis, e.g., lowering effector IL-17C expression reduces infiltrating immune cells and thus inhibits skin inflammation [33,34]. In immunohistochemical experiments, we found that the BT and GT groups significantly inhibited IL-17 expression. It has been shown that ingestion of black tea reduces the expression of the inflammatory cytokine IL-17, which may be attributed to the tea polyphenols it contains [35]. Green tea has a long history of therapeutic use for inflammation and its inhibition of IL-17 has been demonstrated [36]. TNF-α contributes to macrophage and neutrophil activity, stimulates the production of other pro-inflammatory cytokines, and induces the synthesis of reactive oxygen species and lipid peroxidation [37]. In a study on the effect of WT on serum of female rats, it was found that white tea significantly reduced the level of TNF-α compared to the control group [38]. We also found that only the WT group statistically significantly inhibited the expression of TNF-α.

Based on the results of the gene level expression data, the total number of genes detected in the differential groups and the number of significantly up- and down-regulated differential genes, the green tea alcohol extract had the most up- and down-regulated genes, and the white tea alcohol extract had significantly down-regulated genes. The clustering heat map showed that white tea alcohol extract had the best therapeutic effect. From the gene function analysis, GO gene enrichment was mainly shown in signaling receptor activity in the green tea group, REDOX process in the white tea group and cytoskeleton in the black tea group. Gene enrichment of common neuroactive ligand-receptor interaction signaling pathways was closely related to the results of the green and white tea groups. Gene enrichment for cytokine-cytokine receptor interactions and other signaling pathways was closely related to the results of the black tea group.

The macrophage cell line RAW264.7 cells can undergo cellular inflammation induced by LPS, and this cell line has been used extensively to study the anti-inflammatory effects of TEs on skin inflammation at the cellular level. Firstly, the cellular safety of all TEs used was known from the MTT results. Oxidative stress may be one of the reasons for the development of psoriasis [39]. ROS have been reported to damage cellular components and its increased production has been linked to the pathogenesis of psoriasis [40]. Leukocytes infiltrated at inflammatory sites produce ROS through oxidative bursts, which can damage skin cells. Nitric oxide is likewise one of the markers of oxidative stress and is strongly expressed in psoriasis [41]. All TEs significantly reduced the release of ROS and NO, in addition to significant cell migration, suggesting a benefit of antioxidants in the treatment of psoriasis. 

When psoriasis occurs, overexpression of pro-inflammatory cytokines such as IL-1, IL-22, and IFN-γ leads to hyperproliferation of keratin-forming cells [42]. All TEs groups significantly reduced the expression levels of the six selected cytokines, except for the GT group, which had no significant effect on the expression of TGF-β only. TGF-β is a key mediator in the development of inflammation, and it has been shown that BT extract down-regulates TGF-β signaling [43], the same as our findings. One study showed that GT extract downregulated the expression of IL-1β and IL-6, similar to our results [44]. The release of IL-22 exacerbates the inflammatory status of psoriasis skin [45], and in this study, all TEs significantly reduced their expression.

## 4. Materials and Methods

### 4.1. Reagents and Plant Materials

H&E (hematoxylin-eosin) staining, toluidine blue staining, and IHC (immunocytochemistry) kits were from Servicebio (Wuhan, China). The ELISA kits were obtained from Beyotime (Shanghai, China). Tacrolimus cream was purchased from Bausch Health Companies (Quebec, Canada). Imiquimod cream purchased from Zhuhai United Laboratories (Zhuhai, China). Propylene glycol and anhydrous ethanol (superior purity) was obtained from China National Pharmaceutical Group Corporation (Beijing, China). Purified water was obtained from Milli-Q Advantage water purifier (Germany Merck Group Co., Ltd., Darmstadt, Germany). Samples of black tea (Keemun, Anhui), green tea (Hangzhou, Zhejiang) and white tea (Fuding, Fujian) were collected from three local tea factories in southeastern China.

### 4.2. Extraction of Three Alcohol Extracts from Tea

In this experiment, 5 g of dried tea was accurately weighed and crushed, screened for 20 mesh. The maceration method was used for extraction by adding 70% ethanol (as extraction solvent) to the tea leaves in a ratio of 1:30. The extraction time of the tea alcohol extract was 40 min and the extraction temperature was 85 °C. The filtrate was cooled and kept in a volumetric flask. The filtered liquid was transferred to a round bottom flask and evaporated for 30 min at 42–55 °C using a rotary evaporator with controlled air pressure to avoid boiling. A brownish yellow paste extract was obtained. After standing and stratifying, the upper liquid layer was removed and the extract was stored at 4 °C for backup.

### 4.3. Chemical Composition Analysis of Three Kinds of Tea

The compositional analysis of tea ethanol extracts was performed using the instrumental analytical platform: UPLC-Q-Exactive-Orbitrap MS (Thermo, Ultimate 3000LC, Q Exactive HF, sourced from Waltham, MA, USA). Chromatographic column: Zorbax Eclipse C18 (1.8 μm × 2.1 × 100 mm). Chromatographic separation conditions: column temperature 30 °C; The flow rate was 0.3 mL/min, the mobile phase composition was A: water +0.1% formic acid, B: pure acetonitrile, the injection volume was 2 μL, and the temperature of automatic injector was 4 °C. Positive mode: heating temperature 325 °C; Sheath gas flow rate: 45 arb; Auxiliary gas flow rate: 15 arb; Purge gas flow: 1 arb; Electric spray voltage: 3.5 KV; Capillary temperature: 330 °C; S-Lens RF level: 55%. Negative mode: heater temperature 325 °C; Sheath gas flow rate: 45 arb; Auxiliary gas flow rate: 15 arb; Purge gas flow: 1 arb; Electric spray voltage: 3.5 KV; Capillary temperature: 330 °C; S-Lens RF level: 55%. Scanning mode: one-time full scanning (*m*/*z* 100~750) and data-dependent secondary mass spectrometry scanning (dd-MS2, TopN = 10); Resolution: 70,000 (primary mass spectrometry) and 17,500 (secondary mass spectrometry). Collision mode: high energy collision dissociation.

### 4.4. Bacterial Observation and Inhibition Assay

Bacterial morphology was observed using a benchtop scanning electron microscope TM3000 (Hitachi, Tokyo, Japan). *Escherichia coli* (ATCC25922) and *Staphylococcus aureus* (ATCC6538), tested and provided by Guangdong Institute of Microbiology (Guangzhou, China), were selected. Fifty (50) μL of each extract were mixed in bacterial suspensions separately and incubated for 3 h at 30 °C. Another group was added with equal amount of anhydrous ethanol as negative control. At the end of incubation, the bacterial cell pellets were centrifuged and fixed in 2.5% glutaraldehyde solution for 2 h, followed by washing with PBS. Dehydrated with gradients of ethanol (30%, 50%, 70%, 90%, 100%), samples were freeze-dried and observed by gold spray.

### 4.5. Animal Experiments

Thirty SPF (specific pathogen free) KM (Kunming, China) mice (male, 5 weeks old, weighing 34–38 g, purchased from Guangdong Laboratory Animal Center) were kept at 22 °C for 4 days. All animal experimental procedures were approved by the Animal Experimentation Ethics Committee of Guangdong University of Technology (approval code: GDUTXS2022007, approval date: 9 March 2022). The mice were randomly divided into six groups: blank group, model group, positive group, BT group, WT group, and GT group. Psoriasis disease modeling was performed in both the model group and positive group, but the positive group was additionally treated with tacrolimus cream. The cream is a topical ointment used for the treatment of dermatitis-like conditions and has been used extensively in animal models of psoriasis [46,47]. During the modeling stage, mice in all groups except the blank group were coated with imiquimod cream (100 mg/pc) once daily on the back skin for 8 consecutive days. The backs of the mice were depilated with surgical clippers over an area of approximately 4 cm × 4 cm. The experiment was divided into an 8-day modeling period and a 3-day dosing period.

After the model was established, the drug was administered once a day. Propylene glycol solution (200 μL/pc) was applied to the dorsal hair removal site in the model and blank groups, and tacrolimus cream (50 mg/pc) was applied to the posterior hair removal site in the positive group. While in the experimental group, the corresponding drug (200 μL/pc) was applied at the dorsal hair removal site, and the mice in each of the above groups were administered once a day for 3 days. On day 9, the back skin of the mice was observed and PASI scores were recorded to assess epidermal damage. PASI symptoms are measured on a scale of 0 to 4, from none to maximum. The cumulative effect of three scores indicates the severity of inflammation. On day 11, the back skin of the mice was photographed to obtain picture data, and the appearance and PASI scores were recorded and weighed. Mice were then executed for cervical dislocation. The dorsal skin tissues (2.0 cm × 0.8 cm) were fixed with paraformaldehyde and frozen for subsequent experiments.

### 4.6. Histological Observation and Immunohistochemical Analysis

Skin tissues (6 mm) were paraffin-embedded and then stained by H&E to observe the histological changes after extract treatment. After the sections were routinely sectioned, dewaxed in xylene and rehydrated, the sections were stained with hematoxylin and eosin solution, and then the staining was observed under a fluorescent microscope (magnification 200 times). The process of toluidine blue staining is similar to the above, except that the staining solution is toluidine blue.

The expression of TNF-α (Tumor necrosis factor-α) and IL-17 (Interleukin-17) in mouse skin tissues was quantified by immunohistochemistry. Tissue sections were incubated with diluted primary antibody (primary antibody dilution ratio of 1:1000) overnight at 4 °C and then incubated with secondary antibody. Biotin-labeled horseradish peroxidase antibody (secondary antibody at a dilution ratio of 1:3000) was placed at 25 °C for 1 h. Staining was observed and controlled under a microscope (magnification of 200×) using the image analysis software image J version 1.8.0 (developed by the National Institutes of Health). 

### 4.7. Differential Gene Screening and Functional Analysis

The groups of genes were screened with the criterion that the RPKM value of the expression level was greater than 1, which was processed by Pareto conversion. The differentially expressed genes limited threshold was determined by constructing the load correlation coefficient value in the PLS-DA model, and differentially expressed genes screening was combined with Students *t*-test (*p* < 0.05).

Integration of differential genes obtained between different groups. Differential back-regulated genes were analyzed by DAVID database and online web database STRING for network pharmacology. The analysis of these genes and their functional annotation was performed by clustering using GO and KEGG databases.

### 4.8. Cytotoxicity Assay

The cytotoxicity of the three extracts was determined using the 3-(4,5-dimethylthiazol-2-yl)-2,5-diphenyltetrazolium bromide (MTT) colorimetric assay. Briefly, RAW264.7 cells in the exponential growth phase were collected at a cell density of 2 × 10^4^ cells/mL, and inoculated into 96-well plates. Cell suspensions were incubated with 100 μL of tea extract at a concentration of 1%, and equal amounts of medium were added to the blank (medium) and control (cells + medium) groups. After 24 h incubation, the supernatant was removed and 150 μL of dimethyl sulfoxide was added to each well. The solution was shaken well to dissolve for 15 min. Measure the absorbance at 490 nm using a microplate reader (SpectraFluor^®^, Tecan, Zurich, Switzerland). Cell viability = (drug group A value − blank well A value)/(control well A value − blank well A value) × 100%, A is the absorbance value. The data were collated and analyzed for cytotoxic effects of the drugs.

### 4.9. ROS Level Detection

RAW264.7 cells were selected, and cell suspension (2 × 10^4^ cells/mL) was added to each well and incubated for 24 h. After discarding the supernatant, 1 mL of tea extract (1 mg/mL) was added to each well. After 2 h of incubation, lipopolysaccharide (LPS) was added to reach a final concentration of 1 μg/mL. A blank group and an LPS model group were set up, respectively. After continuing incubation for 24 h, the supernatant was discarded and DCFH-DA was added. After 30 min, the supernatant was centrifuged and washed with PBS. Rewire, sample and photograph in an automated fluorescent cell counter.

### 4.10. NO Secretion Detection

RAW264.7 cells were taken and trypsin digestion was performed to adjust the cell concentration. The cell suspension was added to a 96-well cell culture plate and incubated in a CO_2_ incubator for 12 h. The absorbent medium was 190 μL serum-free medium per well. Six hours later, 10 μL samples were added to each group. Samples were added, shaken and mixed on a microplate shaker and the samples were incubated in a CO_2_ incubator for 6, 12, 24 and 48 h. The supernatant was collected and NO content was determined by Griss method. The NO secretion rate was calculated as (sample well OD − blank well OD)/(control well A − control well A) × 100%, A is the absorbance value. 

### 4.11. Cell Scratch Test

Inoculate RAW264.7 cells in the wells and mark the back of the vertical well plate with the gun tip the next day so that the scratch intersects the marker line. Clean the cells to remove the scratched cells. After crossing the line, rinse the cells with sterile PBS to remove the scratched cells and make the gaps left visible to the naked eye. Replace fresh medium with serum-free or low-serum (<2%). Cells were incubated in a 37 °C, 5% CO_2_ incubator. Cells were then removed at the appropriate time and observed under a microscope and photographed for data analysis.

### 4.12. Neutral Red Uptake Assay

Adjust the concentration of RAW264.7 cells to 2 × 10^4^ cells/mL, add 100 μL of bacterial solution to each well of a 96-well plate for 24 h, then discard the supernatant and three kinds of tea extracts with the lowest cytotoxicity (1% 10 mg/mL) with the MTT method were diluted with basic medium and LBS (30 μg/mL) were diluted with basic medium for LBS group. Media containing 30 μg/mL LPS was added to the LPS group, and the corresponding volume of media was added to the blank group. After 24 h of incubation, the supernatant was discarded, washed with PBS, and 100 μL of neutral red solution at a final concentration of 0.1% was added to each well and incubated for 30 min. Stem cells were aspirated and rinsed with PBS. The amount of cell lysate was 100 μL/well. After pyrolysis in a shaker at room temperature for 15 min, the absorbance was measured at 570 nm and the phagocytosis was calculated.

### 4.13. Measurement of Skin IL-1, IL-6, IL-22, IFN-γ, TGF-β and FN Levels

Skin tissue cells of mice in each group were collected, cell medium was prepared, and supernatant was taken. IL-1 (interleukin-1), IL-6 (interleukin-6), IL-22 (interleukin-22), IFN-γ (interferon-γ), TGF-β (transforming growth factor), and FN (fibronectin) were measured according to the ELISA kit instructions.

### 4.14. Statistical Analysis

Data processing and graphing were performed using Graphpad Prism 8.0.2 software. All experimental data were expressed as mean ± standard deviation, and *p* < 0.05 was considered as a significant difference in the results.

## 5. Conclusions

In a mouse model of skin inflammation induced by imiquimod, the anti-psoriasis effect of tea extract was evaluated by measuring its inhibition of several pro-inflammatory cytokines such as IL-1, IL-6, IL-22, FN, IFN-γ, and TNF-α. Histological examination showed that the three groups could effectively eliminate the skin inflammation and cortical hyperplasia caused by imiquimod. Mast cells release a series of allergic mediators through degranulation reaction. All groups can reduce skin inflammation by inhibiting proliferation and infiltration of mast cells and alleviating degranulation phenomenon. IFN-γ can promote transcription, translation and secretion of IL-1, and the three groups can reduce skin inflammatory response by inhibiting IFN-γ-mediated interleukin-immune regulatory pathway. Therefore, these results suggest that all *Camellia sinensis* extracts can resist skin inflammation induced by imiquimod by inhibiting inflammation, among which white tea extract has better anti-inflammatory ability and has potential as an anti-inflammatory material.

## Figures and Tables

**Table 1:** Analytical results of the composition of three tea extracts.

NO.	Identified Compounds ^I^	Relative Content% ^II^			Mass (*m*/*z*)	Formula	Classification ^III^
		BT	GT	WT			
1	Choline	1.67	0.84	1.28	103.09989	C_5_H_13_NO	
2	L-Valine	0.26	0.34	0.34	117.07899	C_5_H_11_NO_2_	a
3	Succinic acid	0.02	0.02	0.03	118.02647	C_4_H_6_O_4_	c
4	4-Hydroxybenzaldehyde	/	0.06	0.02	122.0368	C_7_H_6_O_2_	
5	Nicotinamide	0.18	0.15	0.36	122.04798	C_6_H_6_N_2_O	
6	5-Hydroxymethyl-2-furaldehyde	0.08	0.12	0.08	126.03163	C_6_H_6_O_3_	c
7	*D*-(+)-Pyroglutamic Acid	0.46	0.82	0.50	129.04247	C_5_H_7_NO_3_	c
8	Pipecolic acid	0.08	0.37	0.07	129.07886	C_6_H_11_NO_2_	c
9	L-Norleucine	0.18	0.34	0.27	131.09454	C_6_H_13_NO_2_	c
10	Salicylic acid	0.03	0.05	0.08	138.03149	C_7_H_6_O_3_	c
11	Isophorone	0.12	0.06	0.12	138.10425	C_9_H_14_O	
12	L-Glutamic acid	0.33	0.84	0.44	147.05287	C_5_H_9_NO_4_	a
13	2,3-Dihydroxybenzoic acid	0.02	/	0.02	154.02644	C_7_H_6_O_4_	c
14	4-tert-Amylphenol	0.04	0.06	0.05	164.11852	C_11_H_16_O	p
15	L-Phenylalanine	0.07	0.10	0.08	165.07867	C_9_H_11_NO_2_	a
16	Gallic acid	/	/	0.00	170.02037	C_7_H_6_O_5_	c
17	6,7-Dihydroxycoumarin	0.15	0.02	0.06	178.02626	C_9_H_6_O_4_	s
18	Theobromine	0.21	0.06	0.03	180.06439	C_7_H_8_N_4_O_2_	
19	Citric acid	0.12	0.08	0.23	192.02703	C_6_H_8_O_7_	c
20	Scopoletin	0.01	/	/	192.04196	C_10_H_8_O_4_	
21	*D*-(−)-Quinic acid	3.48	2.68	3.30	192.06323	C_7_H_12_O_6_	c
22	Caffeine	34.37	0.02	17.96	194.07998	C_8_H_10_N_4_O_2_	
23	Gluconic acid	0.36	0.08	0.29	196.05806	C_6_H_12_O_7_	c
24	NP-021797	0.05	/	/	214.1564	C_12_H_22_O_3_	
25	Palmitic acid	/	/	0.01	256.24022	C_16_H_32_O_2_	c
26	Adenosine	0.01	0.01	0.02	267.09582	C_10_H_13_N_5_O_4_	
27	Monolaurin	0.05	0.05	0.08	274.21374	C_15_H_30_O_4_	
28	Linoleic acid	0.02	0.01	0.02	280.24019	C_18_H_32_O_2_	c
29	Elaidic acid	0.12	0.10	0.20	282.25582	C_18_H_34_O_2_	c
30	Stearamide	3.20	5.26	3.24	283.28694	C_18_H_37_NO	
31	Phenylethyl 2-Glucoside	/	0.02	0.01	284.12516	C_14_H_20_O_6_	s
32	Kaempferol	0.02	0.02	0.01	286.04693	C_15_H_10_O_6_	f
33	Luteolin	0.07	0.01	0.01	286.04742	C_15_H_10_O_6_	f
34	9-Oxo-10(E),12(E)-octadecadienoic acid	0.33	0.14	0.31	294.21877	C_18_H_30_O_3_	c
35	Tridemorph	0.20	0.29	0.19	297.3024	C_19_H_39_NO	
36	Morin	0.08	0.10	0.07	302.04181	C_15_H_10_O_7_	f
37	NP-010776	0.07	0.14	0.02	302.04185	C_15_H_10_O_7_	
38	Quercetin	0.03	0.04	0.01	302.04213	C_15_H_10_O_7_	f
39	2,3-dihydroxypropyl 12-methyltridecanoate	1.60	1.32	2.13	302.24482	C_17_H_34_O_4_	
40	(−)-Gallocatechin	0.02	2.87	0.51	306.07345	C_15_H_14_O_7_	p
41	Oleoyl ethylamide	0.79	1.16	0.74	309.30232	C_20_H_39_NO	
42	(±)9-HpODE	/	/	0.01	312.23027	C_18_H_32_O_4_	c
43	(±)9(10)-DiHOME	0.37	0.23	0.53	314.24565	C_18_H_34_O_4_	c
44	NP-008993	0.08	0.06	0.10	314.24587	C_18_H_34_O_4_	
45	NP-020139	/	0.01	0.01	316.07956	C_13_H_16_O_9_	
46	Myricetin	/	0.05	0.02	318.03673	C_15_H_10_O_8_	f
47	5-OxoETE	0.21	0.11	0.24	318.21647	C_20_H_30_O_3_	c
48	Linoleoyl Ethanolamide	0.05	0.06	0.03	323.28161	C_20_H_37_NO_2_	
49	Glabridin	0.07	0.04	0.08	324.1356	C_20_H_20_O_4_	f
50	TOFA	1.39	1.14	1.72	324.22701	C_19_H_32_O_4_	c
51	Corchorifatty acid F	0.03	/	0.01	328.22506	C_18_H_32_O_5_	c
52	NP-002113	0.03	0.02	0.03	332.1956	C_20_H_28_O_4_	
53	Bicyclo Prostaglandin E2	0.09	0.08	0.13	334.21122	C_20_H_30_O_4_	c
54	Erucamide	4.63	4.20	5.89	337.33354	C_22_H_43_NO	
55	NP-000587	0.14	/	0.04	338.1	C_16_H_18_O_8_	c
56	Docosanamide	0.18	0.25	0.21	339.34927	C_22_H_45_NO	
57	Cyanox 2246	6.15	6.88	6.13	340.24028	C_23_H_32_O_2_	p
58	Trehalose	0.82	5.25	2.74	342.11622	C_12_H_22_O_11_	s
59	NP-021781	0.03	0.03	0.05	344.25547	C_19_H_36_O_5_	c
60	Methoxyacetyl fentanyl	0.02	0.01	0.03	352.22175	C_22_H_28_N_2_O_2_	
61	Monoolein	0.61	0.49	0.89	356.29175	C_21_H_40_O_4_	
62	1-Stearoylglycerol	30.98	24.48	40.15	358.30741	C_21_H_42_O_4_	
63	PEG n8	0.12	0.11	0.27	370.21938	C_16_H_34_O_9_	
64	5-(Ethylsulfonyl)-2-[(3*S*)-1-(4-methoxybenzyl)-3-pyrrolidinyl]-1,3-benzoxazole	0.03	0.04	0.03	400.14894	C_21_H_24_N_2_O_4_S	
65	Bis-(4-ethylbenzylidene) sorbitol	2.88	2.79	4.58	414.20329	C_24_H_30_O_6_	
66	AL 8810 Methyl ester	0.02	0.07	0.07	416.24012	C_25_H_33_FO_4_	
67	NP-020760	0.01	0.04	0.02	418.25592	C_21_H_38_O_8_	
69	Isovitexin	0.05	0.03	0.01	432.10491	C_21_H_20_O_10_	s
70	Vitexin	0.04	0.03	0.01	432.10503	C_21_H_20_O_10_	f
71	Afzelin	0.01	/	/	432.1051	C_21_H_20_O_10_	s
72	Nα-({(3*R*,4*R*,5*R*)-4,5-dihydroxy-3-[(3-pyridinylcarbonyl)amino]-1-cyclohexen-1-yl}carbonyl)-L-tyrosinamide	/	27.65	/	440.16493	C_22_H_24_N_4_O_6_	
73	Astragalin	0.13	0.12	0.02	448.1001	C_21_H_20_O_11_	f
74	Oleanolic acid	0.09		0.11	456.35933	C_30_H_48_O_3_	c
75	Epigallocatechin gallate	0.08	3.61	1.07	458.08481	C_22_H_18_O_11_	f
76	Isoquercitrin	0.22	0.62	0.16	464.09494	C_21_H_20_O_12_	s
77	Myricetin 3-*O*-beta-d-galactopyranoside	0.04	0.41	0.14	480.08991	C_21_H_20_O_13_	s
78	PEG n11	0.06	0.05	0.10	502.29807	C_22_H_46_O_12_	
79	Methyl (2*R*,4*S*,6*S*,12b*R*)-4-(4-fluorophenyl)-2-{[2-(4-morpholinyl)ethyl]amino}-1,2,3,4,6,7,12,12b-octahydroindolo[2,3-a]quinolizine-6-carboxylate	0.06	0.04	0.07	506.26366	C_29_H_35_FN_4_O_3_	
80	(3beta,9xi)-3-(beta-d-Glucopyranosyloxy)-14-hydroxycard-20(22)-enolide	0.06	/	0.01	536.2954	C_29_H_44_O_9_	
81	PEG n12	0.04	0.03	0.07	546.32419	C_24_H_50_O_13_	
82	Vicenin III	0.26	0.17	0.08	564.14715	C_26_H_28_O_14_	s
83	Vitexin rhamnoside	0.05	0.07	0.05	578.16279	C_27_H_30_O_14_	s
84	Vicenin II	0.03	0.02	0.01	594.15772	C_27_H_30_O_15_	s
85	Kaempferol-3-*O*-rutinoside	0.08	0.17	0.02	594.15805	C_27_H_30_O_15_	s
86	Rutin	0.15	0.99	0.14	610.15276	C_27_H_30_O_16_	s
87	1,2-Dipalmitoylphosphatidylglycerol	0.45	0.26	0.59	722.51659	C_38_H_75_O_10_P	
88	Grosvenorine	0.01	/	/	740.21585	C_33_H_40_O_19_	f
	Amino acids	0.66	1.29	0.85			
	Carboxylic acids	7.68	6.41	8.22			
	Flavonoids	0.55	4.02	1.29			
	Phenols	6.22	9.81	6.69			
	Sugars	1.84	7.77	3.42			

^I^ Identified chemical compounds according to the data of LC–MS, greater than or equal to 0.01%. Compounds with content greater than or equal to 0.01% were selected. ^II^ The content of compounds identified in the ethanolic extract of tea. ^III^ Classification of identified compounds. a: amino acids; c: carboxylic acids; f: flavonoids; p: phenols; s: sugars.

**Table 2:** The antibacterial test was carried out by disk diffusion method.

Alcohol Extract of Tea	Inhibition Zone Diameter (mm)	
	Gram Negative Bacteria	Gram Positive Microbes
	*Escherichia coli*	*Staphylococcus aureus*
BT	1.9 ± 0.57	3.0 ± 0.20
WT	1.6 ± 0.55	1.5 ± 0.50
GT	1.3 ± 0.81	1.2 ± 0.69
Negative Control	0.9 ± 0.23	1.1 ± 0.34

The diameter of the suppression zone does not include the diameter of the disc (6 mm). No effective antibacterial activity against the tested bacteria or fungi. Data were mean ± standard deviation of three replicates.

## Data Availability

The data presented in this study are available on request from the corresponding author.
